# TTI1 indicates a poor prognosis and is associated with metastasis and immunosuppression of colorectal cancer

**DOI:** 10.1016/j.gendis.2025.101727

**Published:** 2025-06-19

**Authors:** Xiangwen Tan, Yunhua Xu, Shuxiang Li, Qing Fang, Guang Fu, Qiulin Huang, Desi Shang, Kai Fu, Yufei Lv, Shuai Xiao

**Affiliations:** a The First Affiliated Hospital, Cancer Research Institute, Hengyang Medical School, University of South China, Hengyang, Hunan 421001, China; b The First Affiliated Hospital, Department of Gastrointestinal Surgery, Hengyang Medical School, University of South China, Hengyang, Hunan 421001, China; c The First Affiliated Hospital, Department of Clinical Laboratory, Hengyang Medical School, University of South China, Hengyang, Hunan 421001, China; d The First Affiliated Hospital, Cardiovascular Lab of Big Data and Lmaging Artificial Intelligence, Hengyang Medical School, University of South China, Hengyang, Hunan 421001, China; e Institute of Molecular Precision Medicine and Hunan Key Laboratory of Molecular Precision Medicine, Xiangya Hospital, Central South University, Changsha, Hunan 410008, China; f Department of Human Anatomy, Hengyang Medical School, University of South China, Hengyang, Hunan 421001, China; g Hunan Provincial Maternal and Child Health Care Hospital, National Health Commission Key Laboratory of Birth Defect Research and Prevention, Hengyang Medical School, University of South China, Hengyang, Hunan 421001, China

DNA damage and repair encompass a range of alterations and mutations in DNA, serving as a crucial mechanism for maintaining genome stability in tumor cells.^1^ Abnormal expression of oncogenes or tumor suppressors caused by the dysregulation of DNA damage and repair is connected to tumor proliferation, metastasis, and immune microenvironment.^2^ Telomere maintenance 2-interacting protein 1 (TTI1), a regulator of DNA damage and repair, is expressed in numerous organisms and is assumed to play a central role in regulating a wide spectrum of DNA damage responses, telomere maintenance, and checkpoint signaling.^3^ TTI1 was regarded as an oncogene and promoted tumor proliferation in colorectal cancer (CRC), providing preliminary evidence for the significance of TTI1 in CRC.^4^ Nevertheless, the precise role of TTI1 in CRC remains unclear. Therefore, we conducted a comprehensive investigation of TTI1 expression in CRC and its associations with clinical significance, prognosis, molecular mechanisms, tumor immunity, and potential clinical value. In summary, our study identified TTI1's role in CRC and its immune microenvironment, highlighting its significance in metastasis and immunotherapy, with meta-chlorophenylpiperazine (mCPP) identified as a potential modulator.

To elucidate its expression in CRC, we first analyzed TTI1 mRNA expression in tumor and adjacent normal tissues using The Cancer Genome Atlas (TCGA) and Gene Expression Omnibus (GEO) datasets. The results illustrated that TTI1 was significantly overexpressed in CRC tissues (Fig. 1A). This observation was validated in our constructed CRC patient-derived tissue microarrays by immunohistochemistry analysis (Fig. 1B; Fig. S1B) and by quantitative PCR in CRC cell lines (Fig. S1A), which confirmed the consistency between bioinformatics analysis and experimental validation. In addition, we assessed the potential clinical significance of TTI1 in CRC. The diagnostic performance of TTI1 in CRC by receiver operating characteristic curve analysis exhibited a strong diagnostic power, with a value for the area under the curve more than 0.8 across multiple datasets (Fig. S1C). The prognostic value of TTI1 in CRC via Kaplan–Meier curve analysis revealed that high TTI1 expression in CRC patients was significantly associated with poor disease-free survival (Fig. 1C). Above all, these results indicate that TTI1 has a valuable predictive power in the diagnosis and prognosis of CRC.

**Figure 1:**
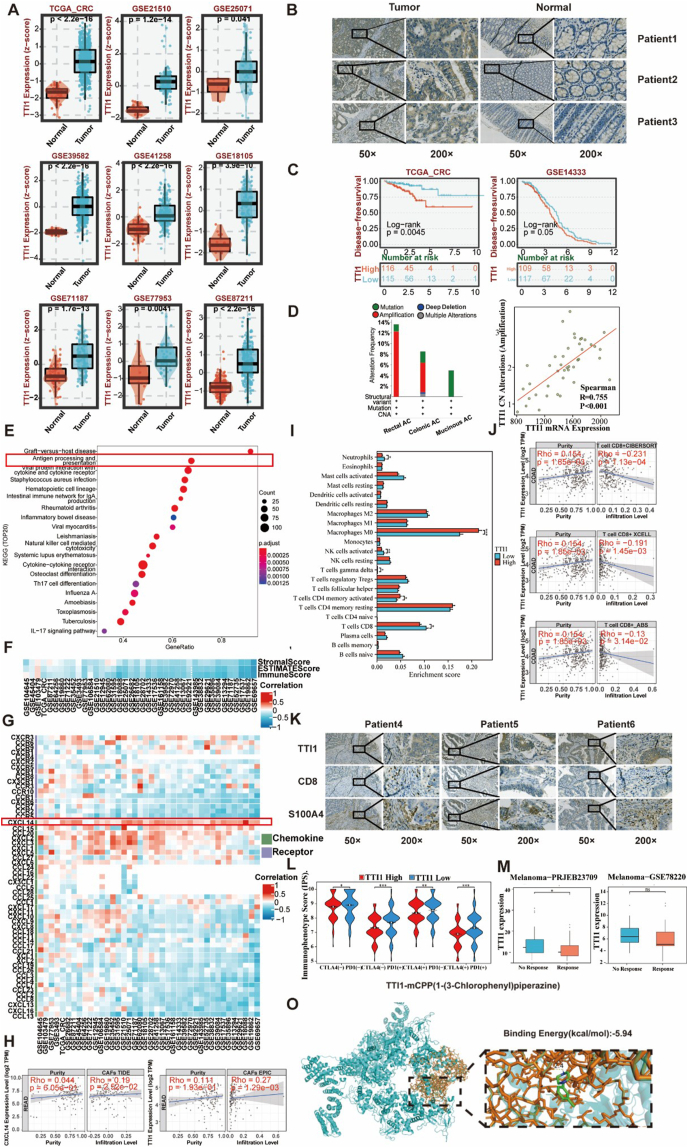
TTI1 drives CRC metastasis and immunosuppression. **(A)** The box plot indicates the difference in TTI1 mRNA expression of tumors and normal tissues across TCGA and GEO. **(B)** The tissue microarray sample demonstrates the differential expression of TTI1 between normal and tumor tissues. **(C)** Kaplan–Meier curves were plotted to predict the TTI1's disease-free survival of TCGA and GSE14333. ∗*p* < 0.05, ∗∗*p* < 0.01, and ∗∗∗*p* < 0.001. **(D)** The genomic alterations of TTI1 in TCGA CRC were analyzed, including mutation, amplification, and deep deletion. The scatter diagram displays the correlation between TTI1 expression and TTI1 copy number alterations (amplification). **(E)** The bar chart represents the KEGG pathways associated with TTI1 by GSEA in CRC. The groups were stratified based on the median expression level of TTI1. **(F)** The heatmap illustrates the correlation between TTI1 expression and immune infiltration score in CRC. **(G)** The heatmap illustrates the correlation between TTI1 expression and chemokines/chemokine receptors in CRC. **(H)** The panel highlights the purity and purity-adjusted correlations between TTI1, CXCL14, and CAFs in CRC. **(I)** The box plot displays the difference in immunocyte infiltration scores between the high and low TTI1 expression groups. **(J)** The panel highlights the purity and purity-adjusted correlations between TTI1 and CD8 in CRC. **(K)** The tissue microarray sample shows the expression of TTI1, CD8, and S100A4. **(L)** The box plot displays the difference in immunophenotype scores (IPS) between the high and low TTI1 expression groups. **(M)** The box plot displays the difference in TTI1 expression between the response and no-response groups. **(O)** Molecular docking between mCPP and TTI1. TTI1, telomere maintenance 2-interacting protein 1; CRC, colorectal cancer; CAF, cancer-associated fibroblast; CXCL14, C-X-C motif chemokine ligand 14; mCPP, meta-chlorophenylpiperazine.

Given that cancer is always characterized by genomic alterations, we next analyzed TTI1 genomic alterations in TCGA CRC samples. Amplification and mutations were the predominant alterations, and further analysis showed that TTI1 amplification was strongly correlated with increased mRNA expression (Fig. 1D). Additionally, TTI1 overexpression was associated with a higher frequency of mutations in CRC-related genes, such as adenomatous polyposis coli (APC) and TP53 (Fig. S2C). Interestingly, TTI1 mutations correlated with reduced mRNA expression, suggesting a functional distinction between amplification and mutation-driven alterations (Fig. S2A, S2B). These findings indicate that TTI1 amplification contributes to its overexpression and may drive CRC progression.

To explore the biological functions of TTI1, we conducted co-expression and functional enrichment analyses. STRING-based protein interaction networks (Fig. S3A) and KEGG pathway analysis (Fig. S3B) revealed TTI1 involvement in autophagy, cellular senescence, and antigen presentation. Gene Set Enrichment Analysis (GSEA) showed that high TTI1 expression was correlated with immunosuppressive processes, including reduced antigen processing and presentation (Fig. 1E; Fig. S3C–S3E), further supporting its potential role in immune regulation.

Considering the role of TTI1 in DNA damage and repair, we further investigated TTI1's correlation with DNA repair mechanisms, including mismatch repair (MMR). Results showed that TTI1 expression was positively correlated with multiple MMR genes (Fig. S4A). The expression of TTI1 was significantly higher in microsatellite stable (MSS) and microsatellite instability-low (MSI-L) subtypes compared with MSI-high (MSI-H) cases (Fig. S4B), supporting its role in immune evasion.^5^ Furthermore, TTI1 expression exhibited a significant negative correlation with tumor mutational burden, MSI, and neoantigen load (Fig. S4C). Since the higher tumor mutational burden, MSI, and neoantigen load always indicate that patients benefit from immunotherapy, these results suggest that TTI1 is likely associated with an immune-excluded tumor microenvironment.

Therefore, we further examined the relationship between TTI1 and immune infiltration. The ESTIMATE (estimation of stromal and immune cells in malignant tumor tissues using expression) algorithm showed that high TTI1 expression was negatively correlated with estimate score, immune score, and stromal score in CRC (Fig. 1F). The antigen presentation-related genes and antigen-presenting cells, including B cells, macrophages, and dendritic cells, were negative correlated with TTI1 expression (Fig. S5A–S5D). These results suggest that TTI1 overexpression potentially suppresses antigen presentation while facilitating immune evasion in CRC.

Since chemokines and chemokine receptors play an important role in immune regulation, we then analyzed their roles with TTI1 expression. Notably, TTI1 expression was negatively correlated with most chemokines and chemokine receptors, except for C-X-C motif chemokine ligand 14 (CXCL14), which was positively correlated (Fig. 1G). High CXCL14 expression was associated with worse recurrence-free survival and overall survival in CRC (Fig. S6A). Given the crucial role of CXCL14 in metastasis, tumor microenvironment, and fibroblasts, we further examined its correlation with cancer-associated fibroblasts (CAFs). The results revealed a strong positive correlation between CXCL14 expression and CAF infiltration (Fig. 1H). Single-cell RNA sequencing analysis confirmed that CXCL14 was primarily expressed in CAFs (Fig. S6B), further suggesting its important role in CRC progression. These findings indicate that TTI1 overexpression may promote CRC metastasis through CXCL14-mediated CAF infiltration.

To further characterize the immune landscape associated with TTI1, we applied the CIBERSORT (cell-type identification by estimating relative subsets of RNA transcripts) algorithm to analyze immune cell infiltration. High TTI1 expression was associated with decreased CD8^+^ T cells and increased CAFs (Fig. 1I; Fig. S7A). Using the TIMER (tumor immune estimation resource) algorithm, we confirmed a significant negative correlation between TTI1 expression and CD8^+^ T cell infiltration, while a positive correlation with CAFs (Fig. 1J). Immunohistochemistry and spatial transcriptomics analysis further validated these findings, showing that high TTI1 expression in CRC tissues was correlated with increased S100A4^+^ CAFs and reduced CD8^+^ T cell infiltration (Fig. 1K; Fig. S7B–S7D). Collectively, these results suggest that TTI1 overexpression contributes to an immunosuppressive tumor microenvironment by enhancing CAF infiltration and inhibiting CD8^+^ T cell-mediated anti-tumor immunity.

To evaluate the clinical implications of TTI1 in immunotherapy response, we used the TIDE (tumor immune dysfunction and exclusion) algorithm. High TTI1 expression was associated with elevated TIDE scores, indicating reduced sensitivity to immune checkpoint blockade therapy (Fig. S8A). Additionally, immunophenotype scoring analysis demonstrated that high TTI1 expression was correlated with lower immunophenotype scores, indicative of reduced tumor immunogenicity (Fig. 1L). In two immunotherapy cohorts (GSE78220 and PRJEB23709), patients with high TTI1 expression exhibited lower response rates to immune checkpoint blockade therapy, with PRJEB23709 showing statistical significance (Fig. 1M). These findings suggest that TTI1 overexpression may serve as a negative predictor of immunotherapy response in CRC.

Finally, we explored the potential TTI1 targeting therapeutic strategies. First, we obtained the 3D structure of the TTI1 protein from the Uniprot and PDB databases, and then retrieved the 3D structure of the top-ranked small molecule compound from the PubChem database. Results showed that mCPP (1-(3-Chlorophenyl) piperazine) was top-ranked among the candidate inhibitors (Fig. S9A, S9B). Finally, molecular docking analysis via PYMOL and AutoDockTools confirmed a stable interaction between TTI1 and mCPP, with a favorable binding energy of −5.94 kcal/mol (Fig. 1O), suggesting the potential of targeting TTI1 by mCPP in CRC.

In summary, the findings imply that TTI1 has an important role in tumor progression, immune suppression, and immunotherapy resistance, highlighting its potential as a biomarker and therapeutic target in CRC. Future studies should focus on elucidating the precise molecular mechanisms underlying TTI1-mediated immune modulation and exploring the clinical utility of TTI1-targeted therapies in CRC patients.

## CRediT authorship contribution statement

**Xiangwen Tan:** Writing – review & editing, Writing – original draft, Visualization, Validation, Software, Methodology, Investigation, Data curation, Conceptualization. **Yunhua Xu:** Writing – review & editing, Supervision, Funding acquisition, Conceptualization. **Shuxiang Li:** Writing – review & editing, Methodology, Conceptualization. **Qing Fang:** Writing – review & editing, Data curation, Conceptualization. **Guang Fu:** Writing – review & editing, Methodology, Data curation, Conceptualization. **Qiulin Huang:** Writing – review & editing, Supervision, Resources, Project administration, Methodology, Conceptualization. **Desi Shang:** Writing – review & editing, Supervision, Resources, Project administration, Methodology, Conceptualization. **Kai Fu:** Writing – review & editing, Supervision, Resources, Project administration, Methodology, Conceptualization. **Yufei Lv:** Writing – review & editing, Supervision, Resources, Project administration, Methodology, Funding acquisition, Conceptualization. **Shuai Xiao:** Writing – review & editing, Supervision, Resources, Project administration, Methodology, Funding acquisition, Conceptualization.

## Ethics declaration

Ethical approval for the experimental procedures involving human tissue samples (tissue microarray and immunohistochemistry) was granted by the Medical Ethics Committee of the First Affiliated Hospital of South China University (Approval Number: 2021LLIY24001).

## Funding

This work was supported by the 10.13039/501100004735Natural Science Foundation of Hunan Province, China (No. 2022JJ30538, 2023JJ60368, 2024JJ6395, 2022JJ50162), Hunan Province Health High-level Talent Scientific Research Project (China) (No. R2023148), and Scientific Research Fund Project of Hunan Provincial Health Commission (China) (No. 202104010105).

## Conflict of interests

The authors declare that the research was conducted in the absence of any commercial or financial relationships that could be construed as a potential conflict of interests.
