# Supplementary material for: TTI1 indicates a poor prognosis and is associated with metastasis and immunosuppression of colorectal cancer
Source: Genes Dis. 2025 Jun 19;13(3):101727. doi: 10.1016/j.gendis.2025.101727 (PMC12854869; doi:10.1016/j.gendis.2025.101727)
Supplement: Multimedia component 1 [file mmc1.docx]

**This file includes:**

**Supplementary Fig. S1 to S9**

**Methods and Materials**

**References**


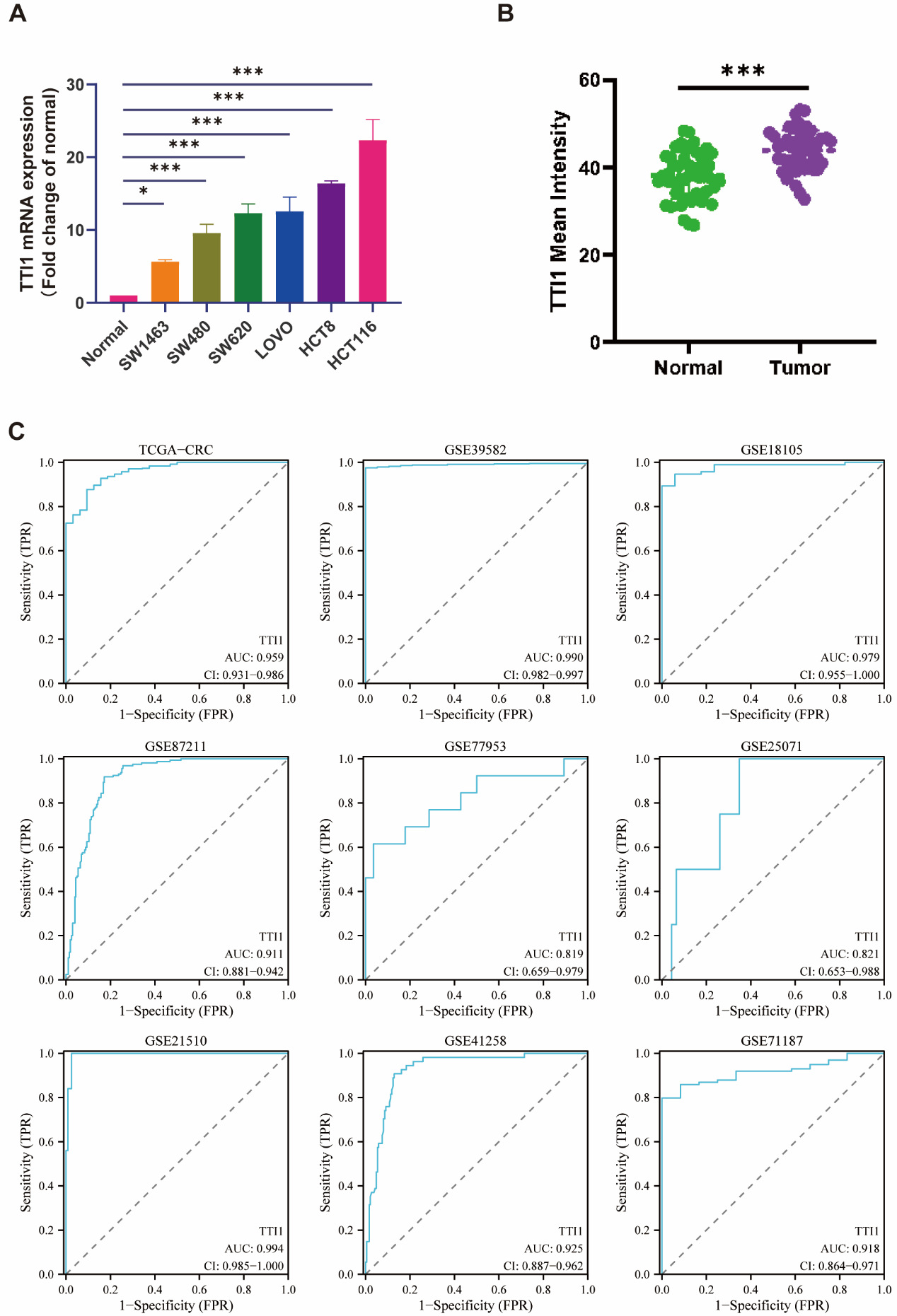


**Figure S1** **TTI1 was highly expressed and served as a biomarker in CRC.** (A) The bar graph illustrates the expression differences of TTI1 between six colon cancer cell lines and normal colon cell lines. (B) The scatter graph showsthe expression differences of TTI1 between tumor and normal tissue. (C) The receiver operating characteristics (ROC) curve shows the diagnostic value of TTI1 expression when comparing patients with tumor and normal tissues.


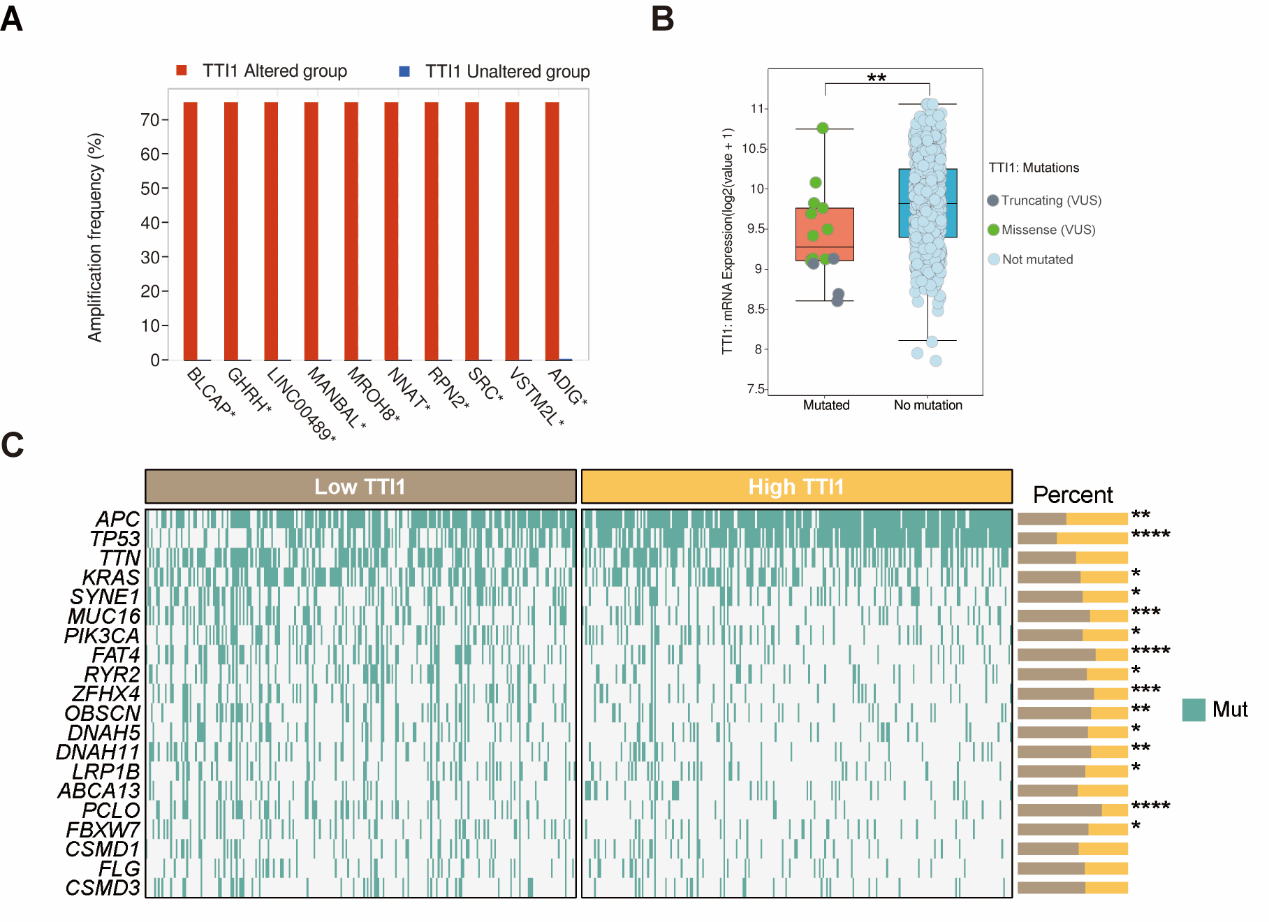


**Figure S2** **Genomic alterations of TTI1 occurred in CRC and exhibited a negative correlation with immune-related markers.** (A) The box plot depicts the difference in TTI1 amplification frequency of altered and unaltered groups. (B) The box plot demonstrates the differential types of mutation between normal and tumor tissues. (C) The waterfall plot shows the difference mutation rate of top 20 mutated genes between TTI1 high expression and TTI1 low expression groups.


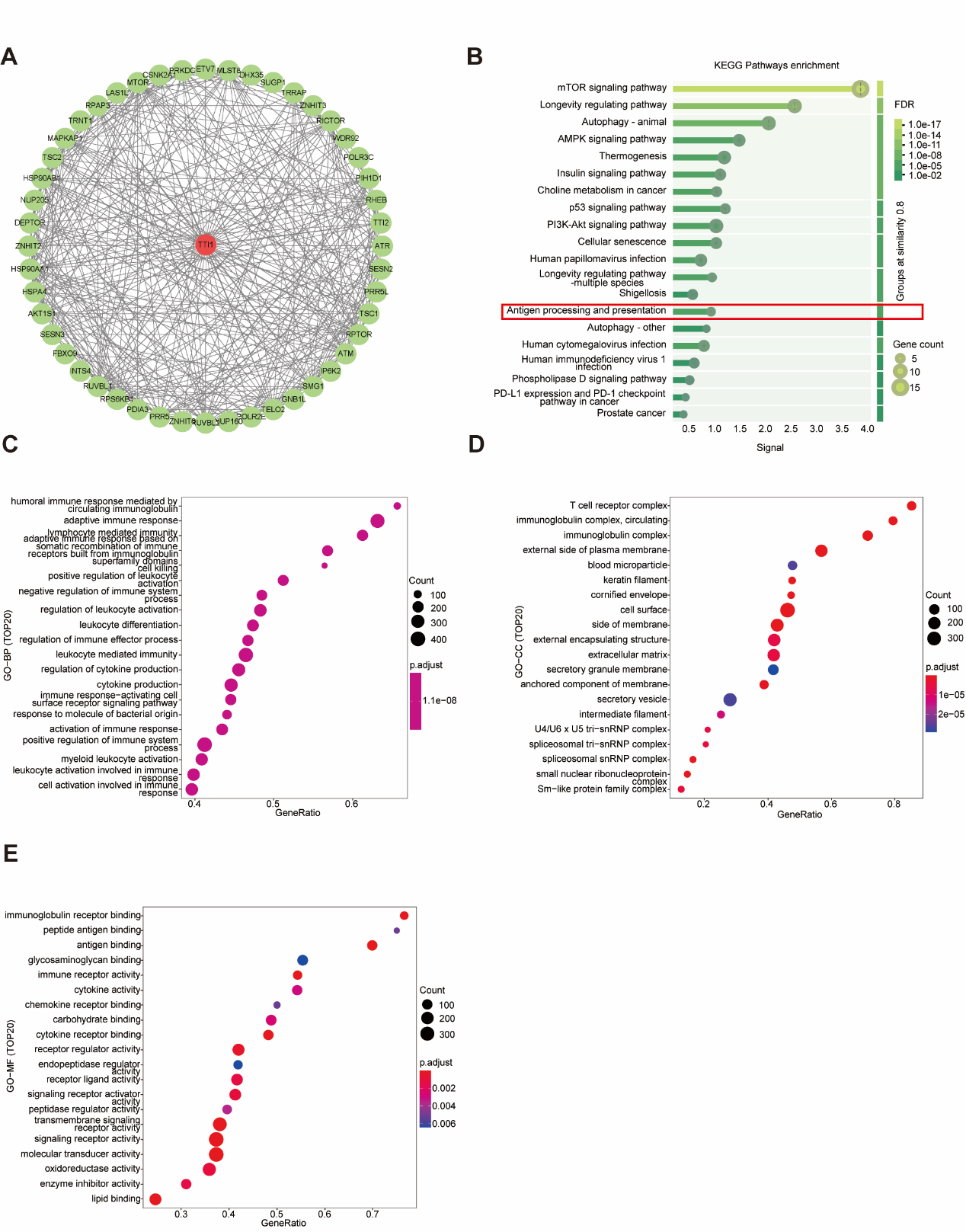


**Figure S3. TTI1 was involved in crucial biological processes in CRC:** (A) Protein–Protein Interaction network of TTI1. (B) The bar chart shows KEGG pathways associated with TTI1 by String in colorectal cancer. (C-E) The bar chart represents the biological processes (BP), cellular components (CC), molecular functions (MF) and associated with TTI1 by GSEA in colorectal cancer. The groups were stratified based on the median expression level of TTI1.


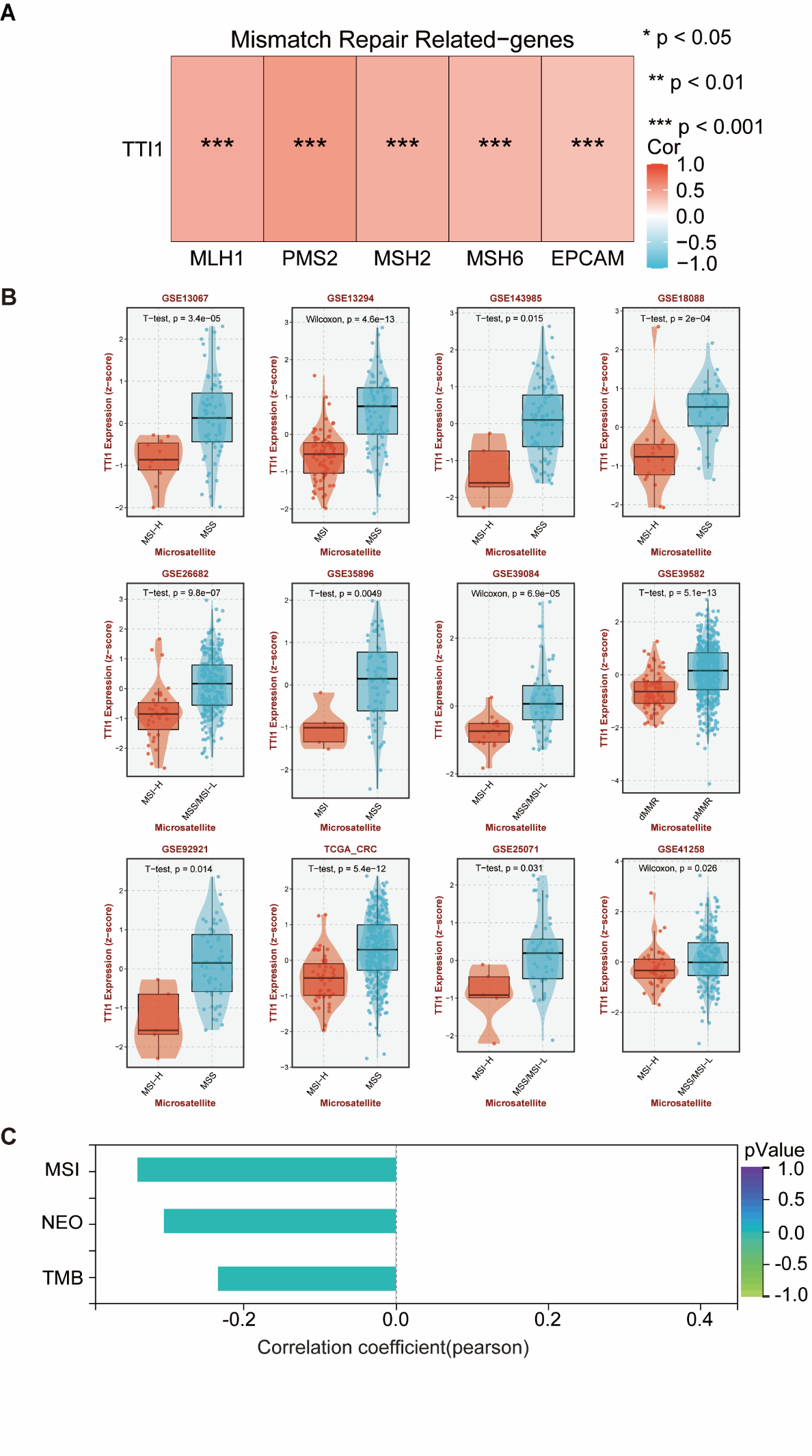


**Figure S4. The TTI1 expression for immunogenicity of CRC**. (A) The heatmap displays the associations between TTI1 and 5 MMR genes in pan-cancer. *, **, and *** represent p < 0.05, p < 0.01, and p < 0.001, respectively. (B) The box plot displays the difference in Microsatellite between the microsatellite stability (MSS), microsatellite instability (MSI-L) low, and MSI-H groups. (C) The bar chart represents the correlation between TTI1 expression and TMB, MSI, NEO.


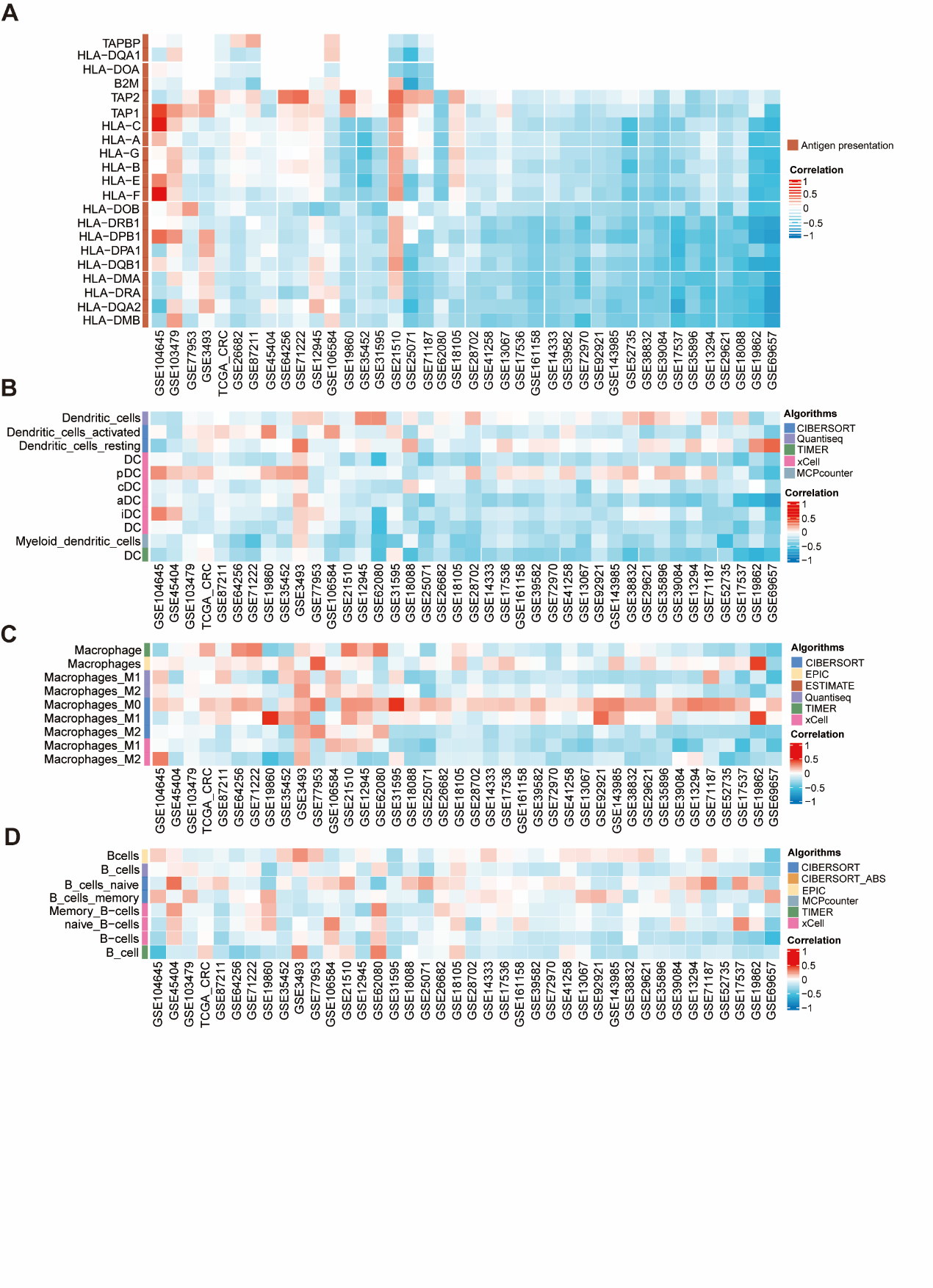


**Figure S5. TTI1 was negatively correlated with immune infiltration and involved in antigen presentation in CRC**. (A) The heatmap depict the correlation between TTI1 expression and antigen presentation-correlated genes in CRC. (B-D) The heatmap shows the correlation between TTI1 expression and antigen presentation-correlated cells (DC, B cell, Macrophages) in CRC.


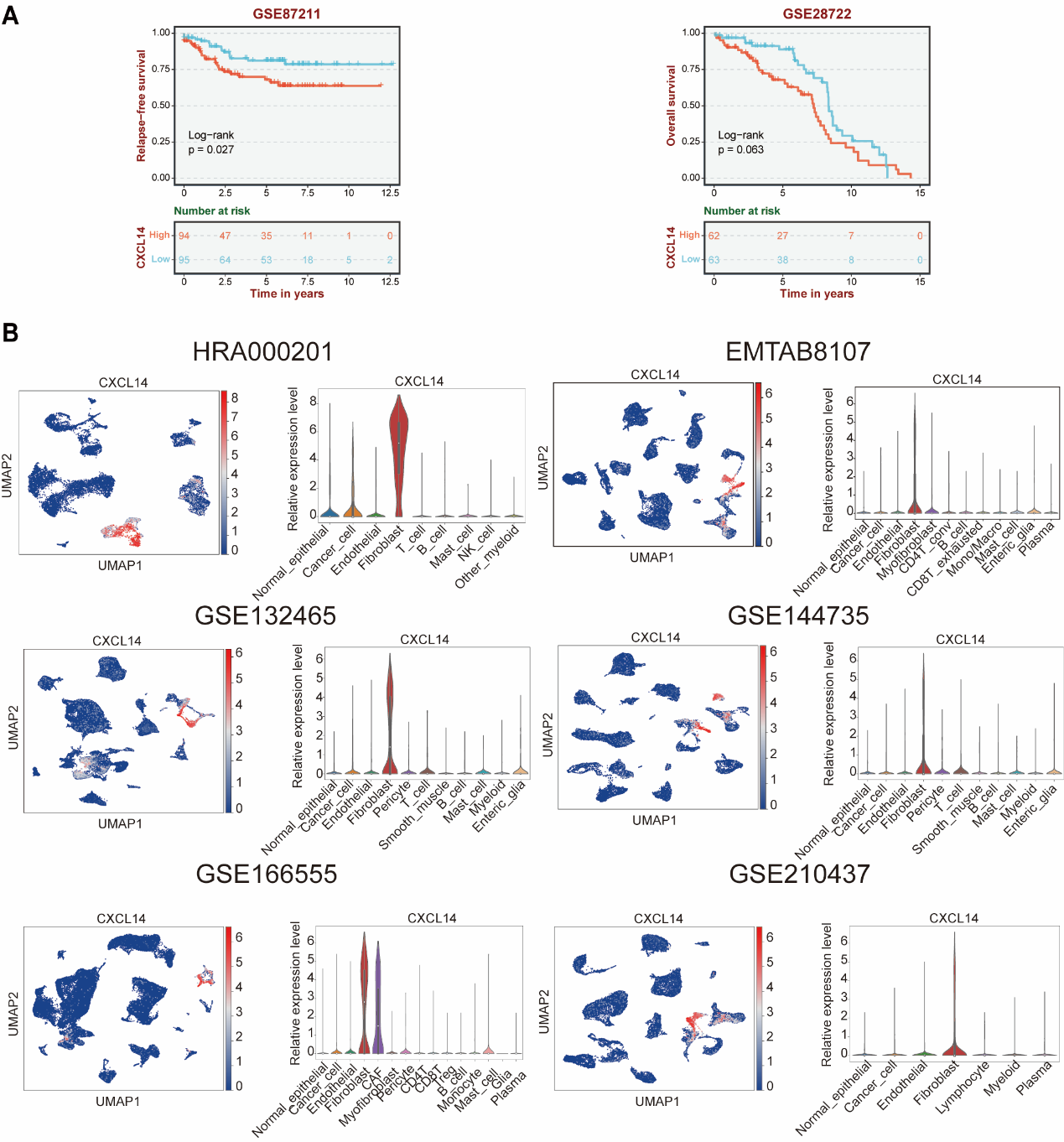


**Figure S6. TTI1 promotes metastasis and CAFs infiltration via CXCL14 in CRC**: (A) Kaplan–Meier curves are plotted to predict the RFS and OS of CXCL14 in GSE87211 and GSE28722 dataset, *, **, and *** represent p < 0.05, p < 0.01, and p < 0.001, respectively. (B) Single cell transcription analysis shows the expression of CXCL14 in CAFs and fibroblasts.


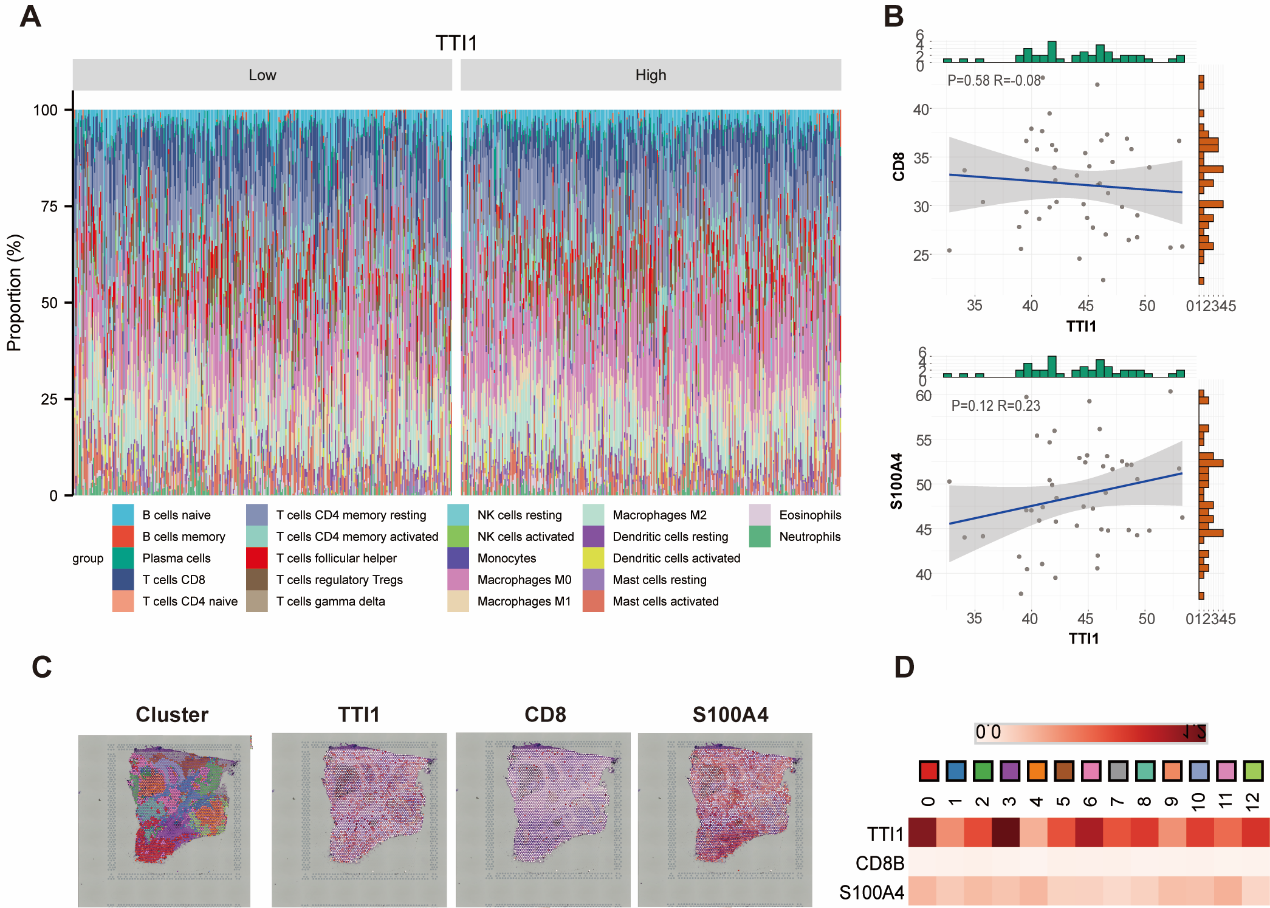


**Figure S7. TTI1 suppresses CD8+ T cell infiltration by CAFs and promotes immune evasion in CRC.** (A) A stacked bar chart shows the difference in immunocyte infiltration scores between the high and low TTI1 expression groups. (B) The scatter plot depicts the correlation between TTI1 and CD8, S100A4. (C) Spatial transcription analysis shows the spatial expression of TTI1, CD8, and CAFs marker. (D)The heatmap depicts the expression of TTI1, CD8, and CAFs marker.


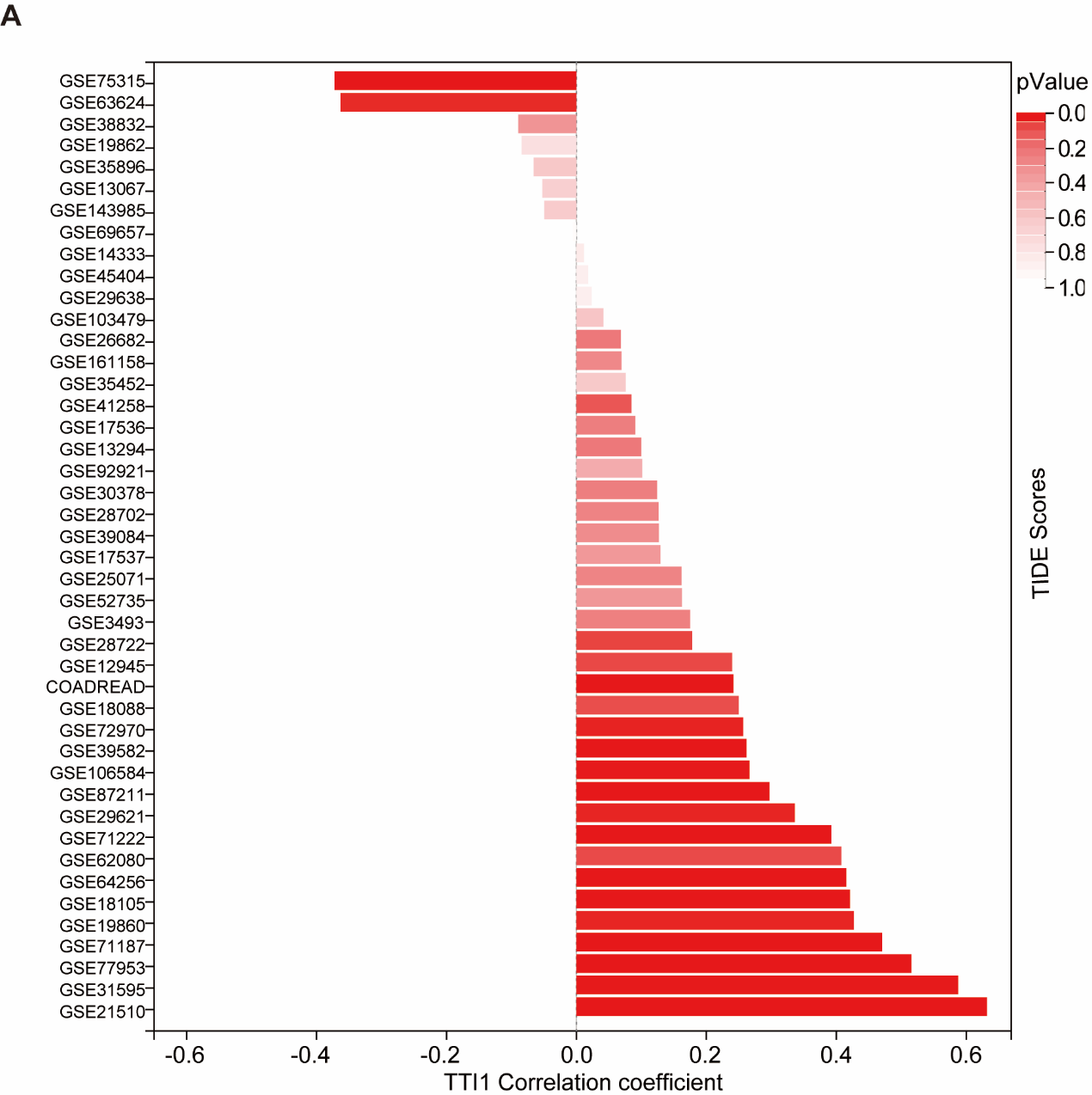


**Figure S8. TTI1 expression negatively predicted the immunotherapy sensitivity of CRC.** (A) The bar chart shows the correlation coefficients between TIDE scores and TTI1 expression in multiple CRC datasets.


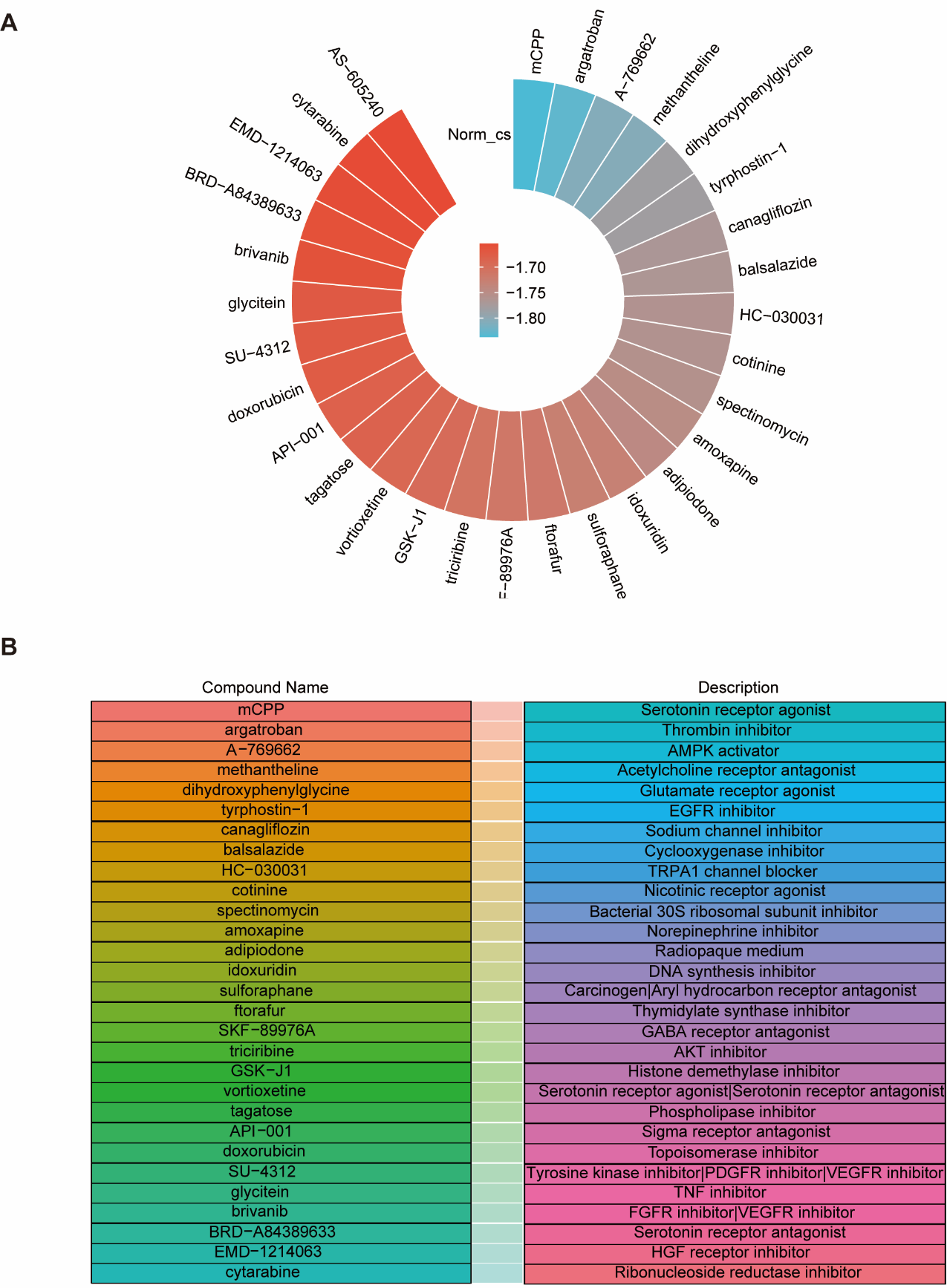


**Figure S9. Identification of mCPP as a potential therapeutic agent targeting TTI1 in CRC**. (A) A circular heatmap plot of Norm connectivity scores across 30 compounds. (B) The description of those top30 compounds.

**MATERIALS AND METHODS**

**Data acquisition and preprocessing**

The RNA sequencing data correspondingclinical information for CRC were collected from multiple sources, including TCGA data portal (https://portal.gdc.cancer.gov/), and GSE3493, GSE10982, GSE12945, GSE13067, GSE13294, GSE14333, GSE17536, GSE17537, GSE18088, GSE18105, GSE19860, GSE19862, GSE21510, GSE25071, GSE26682, GSE28702, GSE28722, GSE29621, GSE29638, GSE30378, GSE31595, GSE35452, GSE35896, GSE38832, GSE39084, GSE39582, GSE41258, GSE45404, GSE52735, GSE62080, GSE62321, GSE63624, GSE64256, GSE69657, GSE71187, GSE71222, GSE72970, GSE75315, GSE77953, GSE87211, GSE92921, GSE103479, GSE106584, GSE143985, and GSE161158 from the Gene Expression Omnibus (GEO)^1^. The obtained datasets were batch-corrected and normalized. The single-cell sequencing datasets of GSE132465, GSE144735, GSE166555, GSE210437, EMTAB8107, HRA000201 form scImmOmics (https://bio.liclab.net/scImmOmics/)^2^. Spatial transcript sequencing datasets of Human Colorectal Cancer: Whole Transcriptome Analysis (Dataset ID: STDS0000033) form stomicsDB (https://db.cngb.org/stomics/)^3^.

**Genomic Alteration Analyses**

The prevalence of genomic alteration types in CRC was scrutinized through the online web tool cBioPortal (https://www.cbioportal.org/)^4^. The "maftools" package was employed to calculate the tumor mutation burden (TMB), microsatellite instability (MSI), and neoantigen data derived from prior research investigations^5-6^. The correlations between TMB, MSI, neoantigen (NEO), and TTI1 expression were then demonstrated. The expression profiling of five major DNA mismatch repair genes (MLH1, MSH2, MSH6, PMS2, and EPCAM) was extracted from CRC samples.

**Prognostic analysis**

The CRC samples were categorized into low and high TTI1 and CXCL14 expression groups using the median TTI1 and CXCL14 expression as the threshold, and Kaplan-Meier survival analysis was performed to study the survival difference. Prognostic risks analyses were performed by the "survival" package in R studios.

**Tissue microarray and immunohistochemistry**

Fresh tumor samples from colorectal cancer (CRC) and the corresponding adjacent non-tumorous colorectal samples were collected with the explicit informed consent of 48 patients undergoing surgical resection. The Medical Ethics Committee of the First Affiliated Hospital of South China University has approved and has obtained informed consent from the patients. The approval number is 2021LLIY24001. The study adheres to the ethical principles outlined in the Declaration of Helsinki (https://www.wma.net/policies-post/wma-declaration-of-helsinki-ethical-principles-for-medical-research-involving-human-subjects/).

**Cell Culture and reverse transcriptase quantitative PCR (RT-qPCR) assay**

The TTI1 mRNA expression level was measured by RT‒PCR assay. Total RNA from HCT116, HCT8, SW1463, LOVO, SW480, and SW620 cells and normal CRC tissues was extracted using RNA extraction kits (Takara) and then reverse transcribed (Takara) according to the manufacturer instructions. These cells were grown in DMEM comprising 10% FBS, 1% streptomycin and penicillin with 37°C.To assess the specificity of each PCR product, a melting curve analysis was performed, and the corresponding cycle threshold (Ct) values were determined. All mRNAs were detected in triplicate, ensuring the reliability of the measurements. The mRNA levels were quantified using the 2^-ΔΔCt method. Statistical significance of the gene expression levels was evaluated using GraphPad Prism 8.0, and differences with a P value < 0.05 were considered to be statistically significant. The primer sequences of TTI1 were as follows: forward primer: 5’-AAGTCATGCTGCGGAACTCA-3’, reverse primer: 5’-GGAACCACTGGGCTAATGCT-3’.

**Gene set enrichment analyses**

The protein‒protein interaction (PPI) network of TTI1 was explored by the online web tool STRING (https://www.string-db.org/)^7^. Gene set enrichment analysis (GSEA) using online web (http://www.linkedomics.org) was used to investigate other biological functions of TTI1^8^. A significance threshold of p < 0.05 was employed.

**Tumor immune microenvironment evaluation**

The functional role of TTI1 in the microenvironment infiltration of CRC was initially explored by computing the Estimation of Stromal and Immune cells scores of tumor tissues on Expression database (ESTIMATE) and evaluating stromal and immune components^9^. The Cibersort, MCPcounter, QuanTIseq, Xcell, EPIC, TIMER, and Cibersort-ABS algorithm was employed to evaluate the immune infiltration levels of TTI1 in CRC. Additionally, the infiltration scores were computed guided by the markers of immune cell types, enabling the examination of differences in infiltration among the high and low TTI1 groups. These calculations were performed by using the R package "ESTIMATE" (version 1.0.13). Additionally, the interconnections of TTI1 expression with cancer-associated fibroblasts (CAFs) and CD8(+) T cells were examined with TIMER2.0^10^. Various immune algorithms were utilized to analyze these associations. The Tumor Immune Dysfunction and Exclusion (TIDE) web tool was used to forecast the potential reactivity of TTI1 to immune response in CRC^11^.

**Molecular docking analysis**

First, we obtained the 3D structure of the TTI1 protein from the Uniprot (https://www.uniprot.org/) and PDB databases. Next, we retrieved the 3D structure of the top-ranked small molecule compound, mCPP (1-(3-Chlorophenyl) piperazine), from the PubChem (https://pubchem.ncbi.nlm.nih.gov/) database. Subsequently, we used PYMOL software to remove water molecules and ligands from the macromolecular protein structure, followed by adding hydrogens to the protein using AutoDockTools software. Finally, molecular docking analysis was performed.

**Statistical Analyses**

The bioinformatics analyses were carried out using RStudio and R 4.2.2. Pearson and Spearman methods were employed to analyze the correlation between variables. Statistical significance was established as a p value below 0.05.

**REFERENCES**

1. Edgar, R., Domrachev, M. & Lash, A. E. Gene Expression Omnibus: NCBI gene expression and hybridization array data repository. Nucleic Acids Res 30, 207–210 (2002).
2. Li, Y.-Y. *et al.* scImmOmics: a manually curated resource of single-cell multi-omics immune data. *Nucleic Acids Res* **53**, D1162-1172 (2024).

3. Xu, Z. *et al.* STOmicsDB: a comprehensive database for spatial transcriptomics data sharing, analysis and visualization. *Nucleic Acids Res* **52**, D1053–D1061 (2024).

4. Cerami, E. *et al.* The cBio cancer genomics portal: an open platform for exploring multidimensional cancer genomics data. *Cancer Discov* **2**, 401–404 (2012).

5. Bonneville, R. *et al.* Landscape of Microsatellite Instability Across 39 Cancer Types. *JCO Precis Oncol* **2017**, PO.17.00073 (2017).

6. Thorsson, V. *et al.* The Immune Landscape of Cancer. *Immunity* **48**, 812-830.e14 (2018).

7. Szklarczyk, D. *et al.* The STRING database in 2021: customizable protein-protein networks, and functional characterization of user-uploaded gene/measurement sets. *Nucleic Acids Res* **49**, D605–D612 (2021).

8. Subramanian, A. *et al.* Gene set enrichment analysis: a knowledge-based approach for interpreting genome-wide expression profiles. *Proc Natl Acad Sci U S A* **102**, 15545–15550 (2005).

9. Yoshihara, K. *et al.* Inferring tumour purity and stromal and immune cell admixture from expression data. *Nat Commun* **4**, 2612 (2013).

10. Li, T. *et al.* TIMER2.0 for analysis of tumor-infiltrating immune cells. *Nucleic Acids Res* **48**, W509–W514 (2020).

11. Fu, J. *et al.* Large-scale public data reuse to model immunotherapy response and resistance. *Genome Med* **12**, 21 (2020).
